# Macular vessel density differs in multiple sclerosis and neuromyelitis optica spectrum disorder: An optical coherence tomography angiography study

**DOI:** 10.1371/journal.pone.0253417

**Published:** 2021-06-17

**Authors:** Małgorzata Rogaczewska, Sławomir Michalak, Marcin Stopa

**Affiliations:** 1 Department of Ophthalmology, Chair of Ophthalmology and Optometry, Poznan University of Medical Sciences, Poznan, Poland; 2 Department of Neurochemistry and Neuropathology, Chair of Neurology, Poznan University of Medical Sciences, Poznan, Poland; Bascom Palmer Eye Institute, UNITED STATES

## Abstract

Multiple sclerosis (MS) and neuromyelitis optica spectrum disorder (NMOSD) are inflammatory and demyelinating diseases that commonly manifest with optic neuritis (ON) but differ in the pathogenic mechanism. Although it was shown that retinal vessels might alter in MS and NMOSD, a comparative study has not been reported. This study evaluated the macular vessel density in 40 MS patients, 13 NMOSD patients, and 20 controls using optical coherence tomography angiography. The vessel density of superficial capillary plexus (SCP) was significantly lower in ON eyes (MS+ON, NMOSD+ON) than in non-ON eyes (MS-ON, NMOSD-ON) and controls. The density of deep capillary plexus (DCP) was significantly increased in MS+ON and MS-ON eyes compared to healthy eyes. In NMOSD+ON and NMOSD-ON, the DCP did not remarkably differ from the control group. A significant positive correlation was noted between SCP and ganglion cell complex (GCC) thickness in MS+ON, MS-ON, and NMOSD+ON. The DCP did not significantly correlate with GCC thickness, but it increased or decreased with ganglion cell loss in MS and NMOSD, respectively. In conclusion, our findings suggest that the capillary changes in MS patients are secondary to ganglion cells’ atrophy, while vasculopathy seems to be a primary process in NMOSD patients.

## Introduction

Multiple sclerosis (MS) and neuromyelitis optica spectrum disorder (NMOSD) are inflammatory and demyelinating diseases of the central nervous system (CNS) [1, 2]. Although optic neuritis (ON) is a common manifestation in both diseases, in NMOSD patients, optic nerve involvement is often bilateral with poorer visual outcomes [3].

In contrast to MS, the disease-specific serum immunoglobulin G exists and can be detected in up to 80% of NMOSD patients [4]. These autoantibodies target the protein aquaporin-4 (AQP4), a water channel presented in the membrane of astrocytes in the CNS. Within the retina, the AQP4 is found in astrocytes and Müller glial cells and strongly expressed in their perivascular and end-foot processes [5, 6]. Retinal capillaries, ensheathed by the membranes of astrocytes and Müller cells, form the inner blood-retinal barrier (iBRB). The disruption of the iBRB allows the anti-aquaporin-4 antibodies (AQP4-IgG) to bind to the water channels and dysregulates retinal water homeostasis [6].

The retinal capillaries can be visualized in vivo using non-invasive optical coherence tomography angiography (OCTA) and expressed quantitatively with a vessel density (VD) parameter. Moreover, OCTA software divides macular vasculature into superficial capillary plexus (SCP) and deep capillary plexus (DCP) [7].

Several studies evaluated the retinal vessel density in MS [8–12] and NMOSD [13–15] patients, but the SCP and DCP were distinguished in three studies concerning multiple sclerosis [8–10], and only in one NMOSD study [14]. However, the results of the studies were not consistent. The common observation was that the SCP was reduced in MS patients when compared with healthy controls [9, 10]. The study of Feucht et al. revealed that lower vessel density of SCP and DCP was associated only with prior ON, but Farci et al. did not find the differences between eyes with or without former optic neuritis [8, 10]. According to Cennamo et al., the ganglion cell complex (GCC) thickness was associated only with SCP [9]. In NMOSD, the vessel density of SCP and DCP was evaluated in the study by Kwapong et al. [14]. They reported that the capillary reduction of both plexuses was noted in NMOSD eyes when compared with controls [14].

It is still a matter of debate whether the reduction of retinal vessels is primary or secondary to ganglion cells loss [8–10, 13–15]. Anatomically, the DCP is supplied by vertical anastomoses from the SCP; thus, the reduction of the SCP should influence the DCP [7]. In the case of primary retinal vasculopathy, the vessel loss of both plexuses should be observed. Regarding the ganglion cells damage as a causative mechanism, the plexus supplying this retinal layer, i.e., SCP, due to the decreased cell metabolic demand, should be more affected than DCP. In view of the distinct pathogenic mechanism of MS and NMOSD, the comparative characteristics of superficial and deep vascular plexuses may clarify this issue. Such an analysis was not previously reported.

In this study, we aimed to identify macular perfusion abnormalities in patients with MS and NMOSD by using OCTA. Furthermore, we evaluated the SCP and DCP in relation to GCC thickness.

## Materials and methods

### Study participants

In this observational study, patients with multiple sclerosis and patients with AQP4-IgG seropositive NMOSD were recruited from the Department of Ophthalmology and the Department of Neurology of the Poznan University of Medical Sciences between June 2018 and September 2020. All patients with MS fulfilled the revised 2017 McDonald criteria [16]. Clinical data, including disease duration, age at disease onset (defined as years since first symptoms), number of ON attacks, and ongoing therapy, were recorded. The enrolled eyes of MS and NMOSD were divided into subgroups: eyes with a history of optic neuritis (MS+ON, NMOSD+ON) and eyes with no history of ON (MS-ON, NMOSD-ON). Age- and sex-matched healthy volunteers served as controls. The selected participants cohorts were also evaluated by our group in the other study of the peripapillary neurovascular alterations [17].

Anti-aquaporin-4 antibodies were detected by means of indirect fluorescence using a commercial cell-based assay with aquaporin 4 transfected cells (EUROIMMUN AG, Lübeck, Germany). Analyzes were performed in the Department of Neurochemistry and Neuropathology at Poznan University of Medical Sciences, which participates in an international external quality control system and receives regular certification for the detection of AQP4-IgG (Institut für Qualitätssicherung, Lübeck, Germany).

The examination protocol included best-corrected visual acuity (BCVA) measurement, Goldmann applanation tonometry (adjusted for central corneal thickness), slit-lamp biomicroscopy, indirect ophthalmoscopy, spectral-domain OCT (SD-OCT), and OCT angiography. BCVA was assessed with the Early Treatment of Diabetic Retinopathy Study chart and expressed as logMAR.

Eligibility criteria were age ≥ 18 years, no ON attack within 6 months prior to enrollment, and at least 2 years of disease duration for MS patients. We excluded participants with myopia > 6 diopters, macular disease, hypertensive or diabetic retinopathy, glaucoma, history of uveitis or eye surgery, and low OCT image quality.

The research was performed in accordance with the Declaration of Helsinki and was approved by the medical ethics committee of Poznan University of Medical Sciences (an approval no. 562/18 from May 2018). Written informed consent was obtained from each participant after an explanation of the nature of this study.

### SD-OCT

The ganglion cell complex (GCC) thickness was obtained with RTVue XR Avanti with AngioVue (Optovue Inc., Fremont, CA, USA; software version 2017.1.0.151). The GCC scan, which covers a 7 x 7 mm area of the macula, was centered 1 mm temporal to the fovea. The device automatically measured GCC thickness from the internal limiting membrane (ILM) to the outer boundary of the inner plexiform layer (IPL).

### OCT angiography

OCTA is based on a split-spectrum amplitude-decorrelation angiography algorithm, which detects the motion of erythrocytes in the vessels through sequentially obtained OCT cross-sectional scans. The generated blood flow map presented the vessel density (VD), i.e., the percentage area occupied by the perfused retinal blood vessels in the analyzed region [18, 19].

The OCTA image acquisition was performed with RTVue XR Avanti with AngioVue (Optovue Inc., Fremont, CA, USA; software version 2017.1.0.151). Macular vessel density was visualized using a 3 x 3 mm scan centered on the foveola. The AngioVue software automatically segmented the 3-dimensional image of the inner retinal capillaries into two plexuses: the superficial capillary plexus comprising the vessels of the nerve fiber layer (NFL), the ganglion cell layer (GCL), and the inner plexiform layer (IPL); and the deep capillary plexus consisting of the inner nuclear layer (INL) and the outer plexiform layer (OPL) vasculature. The whole vessel density of SCP and DCP were taken into analysis. In addition, to evaluate the association between the plexuses in relation to the GCC thickness, we calculated the difference between DCP and SCP, hereinafter called "the discrepancy between DCP and SCP".

The low-quality images with the signal strength index < 50 or significant motion artifacts were not analyzed. The OCT data were acquired and reported in alignment with APOSTEL recommendations [20].

### Statistical analysis

Statistical analysis was performed using Statistica v13.1 (StatSoft, Inc., Tulsa, USA) and SPSS (SPSS, Inc., Chicago, USA). The distribution of continuous variables was evaluated using the Shapiro–Wilk test. Differences among the cohorts were tested using the Chi-square test for sex and the Kruskal-Wallis test for age and BCVA. To account for intrasubject inter-eye dependencies, we used generalized estimating equation models for comparison of SD-OCT and OCTA parameters between cohorts. Pearson correlation coefficients were calculated to assess the association between OCTA parameters and GCC thickness. Due to the exploratory nature of this study, no adjustment for multiple comparisons was made. Statistical significance was set at *p* < 0.05.

## Results

### Study population

In total, 40 patients with MS, 13 patients with NMOSD, and 20 healthy controls were enrolled into this study. We excluded 3 eyes of MS patients due to poor fixation and 9 eyes of NMOSD patients due to poor fixation (n = 7), cataract (n = 1), and chorioretinal scar (n = 1). The number of eyes with a history of optic neuritis in MS and NMOSD patients was 31 and 8, respectively. At baseline, there were no significant differences between patients and controls on age, sex, and BCVA of enrolled eyes. Demographic and clinical features are summarized in Table 1.

**Table 1:** Demographic and clinical characteristics of MS, NMOSD patients, and controls.

	MS	NMOSD	Controls
Number of subjects	40	13	20
Number of eyes enrolled	77	17	40
ON+	31	8	-
ON-	46	9	40
Age (years), mean ± SD	35.15 ± 7.47	42.08 ± 10.23	37.90 ± 11.47
Sex (female/male)	32/8	11/2	17/3
Age at disease onset (years), mean ± SD	24.30 ± 6.53	30.85 ± 7.22	-
Disease duration (years), median (min-max)	8 (3–32)	9 (1–33)	-
BCVA of enrolled eyes (logMAR), median (min-max)	0.00 (0.00–0.20)	0.00 (0.00–0.20)	0.00 (0.00–0.00)

*BCVA*, best-corrected visual acuity; *logMAR*, the logarithm of the minimum angle of resolution; *max*, maximum; *min*, minimum; *MS*, multiple sclerosis; *NMOSD*, neuromyelitis optica spectrum disorder; *ON*, optic neuritis; *SD*, standard deviation.

### SD-OCT

**Fig 1:**
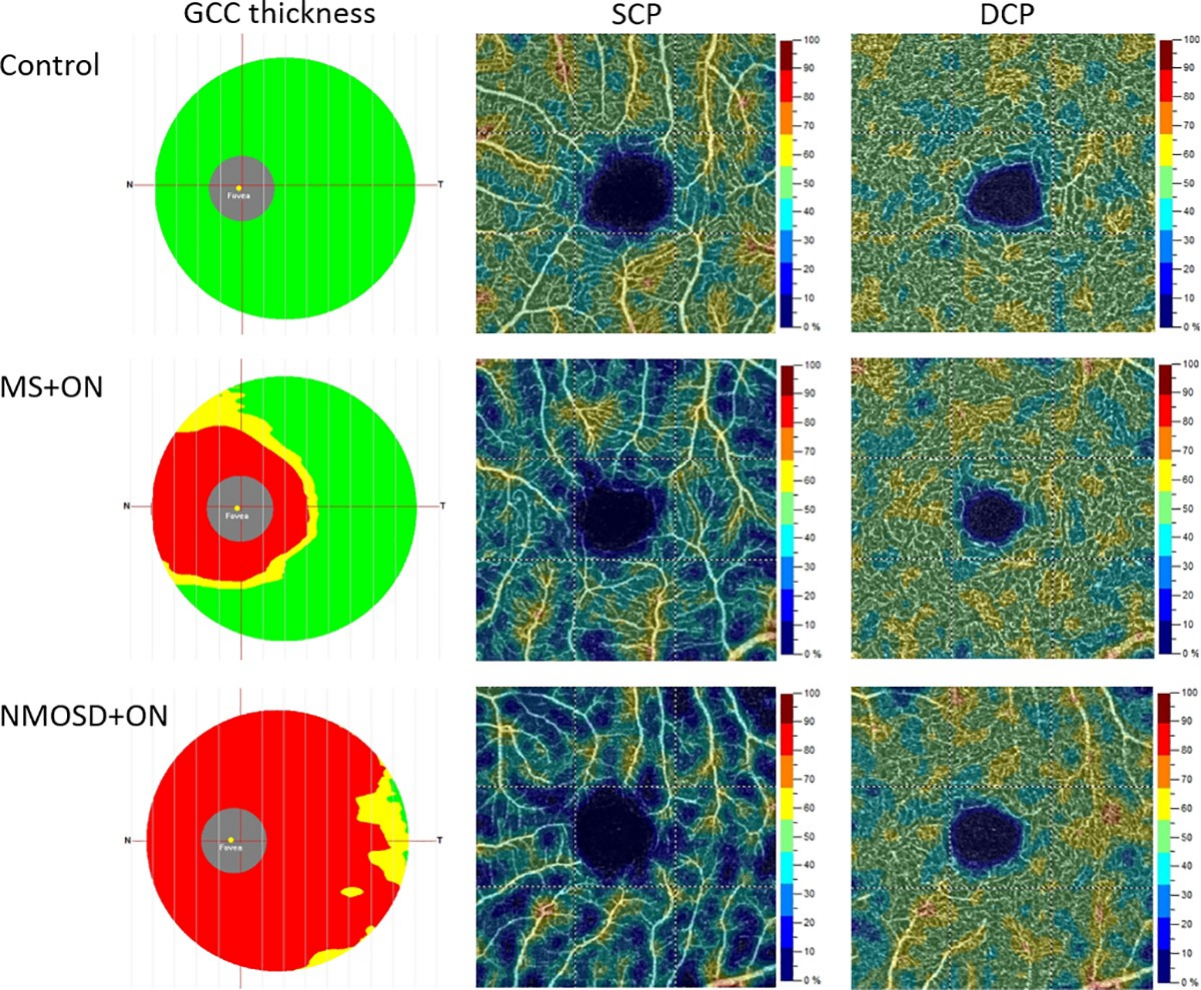
Representative spectral-domain OCT and OCT angiography images of the healthy, MS+ON, and NMOSD+ON eyes. The eyes with a history of optic neuritis present the ganglion cell complex thinning, more severe in NMOSD+ON eye. Compared with controls, the color-coded flow density maps show a diffuse reduction in superficial capillary plexus, more marked in NMOSD+ON than in MS+ON. The vessel density of the deep capillary plexus is higher in MS+ON than in NMOSD+ON and healthy eye. *MS+ON*, multiple sclerosis with optic neuritis; *NMOSD+ON*, neuromyelitis optica spectrum disorder with optic neuritis; *OCT*, optical coherence tomography.

The average ganglion cell complex thickness was significantly lower in the MS+ON, MS-ON, and NMOSD+ON groups than in the controls (*p* < 0.001; Tables 2 and 3; Fig 1). A significant difference in GCC thickness between eyes with or without ON was observed among patients with the same diagnosis (Table 4). However, regarding the same eye status (ON+ or ON-) between MS and NMOSD patients, the thickness was comparable in these groups (*p* > 0.05; Table 4).

**Table 2:** Baseline spectral-domain OCT and OCT angiography results of patients and controls.

Groups	No. of eyes analyzed	Vessel density (%)			GCC (μm)
		SCP Mean ± SD	DCP Mean ± SD	DCP-SCP Mean ± SD	Average Mean ± SD
**MS+ON**	31	41.03 ± 4.02	54.91 ± 2.29	13.88 ± 4.67	83.61 ± 8.66
**MS-ON**	46	44.0 ± 2.70	53.97 ± 2.22	9.97 ± 3.20	90.74 ± 7.72
**NMOSD+ON**	8	39.61 ± 6.02	53.39 ± 2.18	13.78 ± 6.14	77.75 ± 10.01
**NMOSD-ON**	9	45.08 ± 2.96	53.01 ± 1.32	7.96 ± 2.93	94.33 ± 8.58
**Controls**	40	46.96 ± 2.31	52.19 ± 2.70	5.22 ± 2.58	99.15 ± 5.05

*DCP*, deep capillary plexus; *GCC*, ganglion cell complex; *MS*, multiple sclerosis; *NMOSD*, neuromyelitis optica spectrum disorder

*OCT*, optical coherence tomography; *ON*, optic neuritis; *SCP*, superficial capillary plexus; *SD*, standard deviation.

**Table 3:** Differences in vessel density and ganglion cell complex thickness between patients and controls.

	**MS+ON vs controls**			**MS-ON vs controls**		
	**β**	**95% CI**	**p-value**	**β**	**95% CI**	**p-value**
**SCP**	-5.937	(-7.783 to -4.090)	<0.001	-2.960	(-4.141 to -1.780)	<0.001
**DCP**	2.725	(1.399 to 4.050)	<0.001	1.787	(0.568 to 3.006)	0.004
**DCP-SCP**	8.661	(6.500 to 10.823)	<0.001	4.747	(3.291 to 6.203)	<0.001
**GCC**	-15.537	(-19.581 to -11.493)	<0.001	-8.411	(-11.915 to -4.907)	<0.001
	**NMOSD+ON vs controls**			**NMOSD-ON vs controls**		
	**β**	**95% CI**	**p-value**	**β**	**95% CI**	**p-value**
**SCP**	-7.350	(-11.634 to -3.066)	<0.001	-1.885	(-4.517 to 0.748)	0.161
**DCP**	1.203	(-0.581 to 2.986)	0.186	0.848	(-0.548 to 2.245)	0.234
**DCP-SCP**	8.553	(4.239 to 12.866)	<0.001	2.733	(-0.063 to 5.529)	0.055
**GCC**	-21.400	(-28.581 to -14.219)	<0.001	-4.817	(-12.459 to 2.826)	0.217

*β*, regression coefficient; *CI*, confidence interval; *DCP*, deep capillary plexus; *GCC*, ganglion cell complex; *MS*, multiple sclerosis; *NMOSD*, neuromyelitis optica spectrum disorder; *ON*, optic neuritis; *SCP*, superficial capillary plexus.

**Table 4:** Comparison of vessel density and ganglion cell complex thickness between selected groups.

	**MS+ON vs MS-ON**			**NMOSD+ON vs NMOSD-ON**		
	**β**	**95% CI**	**p-value**	**β**	**95% CI**	**p-value**
**SCP**	-2.976	(-4.787 to -1.166)	<0.001	-5.465	(-10.339 to -0.591)	0.028
**DCP**	0.938	(-0.128 to 2.004)	0.085	0.354	(-1.385 to 2.093)	0.690
**DCP-SCP**	3.914	(1.792 to 6.036)	<0.001	5.819	(0.906 to 10.733)	0.020
**GCC**	-7.126	(-11.543 to -2.710)	0.002	-16.583	(-26.626 to -6.541)	0.001
	**NMOSD+ON vs MS+ON**			**NMOSD-ON vs MS-ON**		
	**β**	**95% CI**	**p-value**	**β**	**95% CI**	**p-value**
**SCP**	-1.413	(-5.912 to 3.085)	0.538	1.076	(-1.532 to 3.683)	0.419
**DCP**	-1.522	(-3.205 to 0.160)	0.076	-0.938	(-2.092 to 0.215)	0.111
**DCP-SCP**	-0.109	(-4.691 to 4.473)	0.963	-2.014	(-4.780 to 0.752)	0.153
**GCC**	-5.863	(-13.531 to 1.805)	0.134	3.594	(-4.252 to 11.440)	0.369

*β*, regression coefficient; *CI*, confidence interval; *DCP*, deep capillary plexus; *GCC*, ganglion cell complex; *MS*, multiple sclerosis; *NMOSD*, neuromyelitis optica spectrum disorder; *ON*, optic neuritis; *SCP*, superficial capillary plexus.

### OCTA

The vessel density of SCP was significantly lower in MS+ON, MS-ON, and NMOSD+ON when compared with controls (*p* < 0.001) and in ON eyes of MS and NMOSD groups when compared with non-ON eyes (Tables 2–4; Fig 1). On the contrary, the density of DCP was significantly increased in MS+ON and MS-ON eyes compared to control eyes (*p* < 0.001 and *p* = 0.004, respectively). The NMOSD patients, with or without ON, have similar DCP parameters to controls (Table 3).

### The discrepancy between DCP and SCP

**Fig 2:**
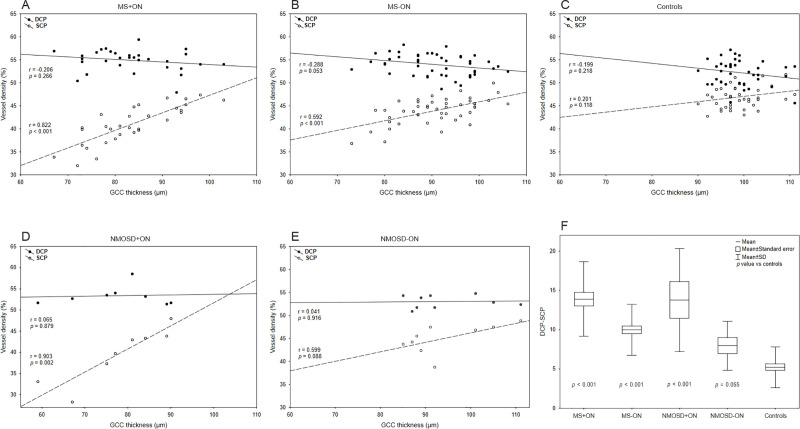
Graphical presentation of spectral-domain OCT and OCT angiography results. Scatter plots illustrating the linear association between GCC thickness and vessel density of SCP and DCP in patients and controls (A-E). The box plot presents the discrepancy between DCP and SCP of all groups (F). *DCP*, deep capillary plexus; *GCC*, ganglion cell complex; *OCT*, optical coherence tomography; *SCP*, superficial capillary plexus.

The difference between the deep and superficial capillary plexuses (DCP-SCP) was significantly higher in MS+ON, MS-ON, and NMOSD+ON than in controls (*p* < 0.001; Table 3; Fig 2F) and in ON eyes of MS and NMOSD patients as compared to the non-ON eyes (*p* < 0.001 and *p* = 0.020, respectively; Table 4).

### Association of OCTA and SD-OCT

A significant positive correlation was observed between SCP and GCC thickness in MS+ON, MS-ON, and NMOSD+ON groups (*p* < 0.001; Fig 2A, 2B and 2D). Although the vessel density of DCP was not significantly correlated with GCC thickness, the DCP of MS and NMOSD patients tended to increase or decrease with ganglion cell loss, respectively (Fig 2A, 2B, 2D and 2E). The DCP-SCP strongly negatively correlated with GCC thickness in MS+ON, MS-ON and NMOSD+ON patients (r = -0.81, *p* < 0.001; r = -0.699, *p* < 0.001; r = -0.863, *p* = 0.006, respectively).

## Discussion

In this study, we used OCTA to evaluate the macular vessel density in superficial and deep capillary plexuses in patients with MS and NMOSD. We found that SCP and GCC thickness were significantly lower in ON eyes of MS and NMOSD patients than in the controls, and the parameters strongly correlated to each other. Notably, such association was also observed in MS-ON eyes, indicating that former ON was not obligatory to cause ganglion cell loss and SCP reduction in MS cohort.

In MS patients, the SCP’s vessel density was more decreased in eyes with previous ON than in non-ON eyes. On the contrary, the DCP did not differ between ON+ and ON- eyes but had significantly higher vessel density than controls. Although the DCP did not correlate with GCC thickness, the negative trend could be easily observed (Fig 2A and 2B). Moreover, the discrepancy between SCP and DCP was strongly negatively correlated with GGC thickness and was significantly higher in ON eyes.

Nesper et al. reported that retinal vessels could actively adapt to the metabolic demand of retinal cells. The coupling mechanism allows for regulation of the retinal blood flow between plexuses, and, e.g., the higher DCP perfusion may result from vessel dilation or increased velocity of flow [21]. According to these observations, the relationship between SCP and DCP in MS eyes can be explained. SCP and DCP, respectively, supply the ganglion cell layer and inner nuclear layer. It was shown that after optic neuritis, the GCL becomes atrophic, whereas the INL remains unchanged, or its volume increases [22, 23]. It was consistent with our findings that the reduced vessel density was observed only in SCP because of the lower metabolic demand of injured ganglion cells. Additionally, the redistribution of blood between plexuses through vertical anastomoses resulted in an increased density of DCP. Our study’s results nicely demonstrated that the reduction of vessel density in SCP is secondary to ganglion cell loss in MS+ON and MS-ON eyes.

Contrary to the MS-ON group, the NMOSD-ON eyes had comparable SCP, DCP, and GCC parameters to controls. In the studies of Huang et al. and Chen et al., the vessel density was measured only in SCP, and in NMOSD+ON and NMOSD-ON patients, they found it lower than in healthy eyes [13, 15]. Moreover, the authors suggested that it might be evidence for subclinical primary retinal vasculopathy [13, 15]. In our NMOSD patients, the only significant difference in vessel density was noted in SCP between NMOSD+ON eyes and controls. In accordance with other studies, the GCC thickness was significantly lower in ON eyes than in healthy eyes, and in non-ON eyes, it was similar to controls [13, 15].

Comparing the features of MS and NMOSD patients, we made an interesting observation. Although the SCP tended to decrease with ganglion cell loss in both groups, the DCP behaved differently, which is well seen on the plots (Fig 2A and 2D). While the DCP of MS eyes inclined to increase, in NMOSD eyes, it had a tendency to decrease with ganglion cell loss slightly. It shows that the blood flow distribution pattern between plexuses in NMOSD differed from the previously described in MS. The increasing trend of DCP with GCC thickness loss was also observed in controls (Fig 2C), and we can assume that the typical network of healthy vessels can adapt and act this way. Thus, the lack of a proportional increase in DCP’s vessel density may indicate the capillary loss in NMOSD eyes. It also suggests that vascular abnormalities may appear prior to optic neuritis, as Huang et al. and Chen et al. reported [13, 15]. Inconsistent results were published on hemodynamics in the optic neuritis eye vasculature using ultrasound examination [24, 25]. There are reports on the effects of the upregulation of Th17 cells that transforming growth factor-beta, which can cause myointimal fibrosis in NMO patients [26]. Moreover, the vascular reactivity, e.g., effects of nitric oxide, should be considered during the interpretation of results. However, no disturbances in cerebrovascular reactivity in MS patients were recently reported [27].

The retinal capillary network evaluation showed the difference between superficial and deep capillary plexuses in MS and NMOSD patients. The compensatory vascular mechanism of DCP was only seen in MS eyes, indicating that the vessel density of DCP in NMOSD patients was reduced. We think that the explanation may be found in the distinct pathogenic mechanism of NMOSD. Retinal capillaries of SCP and DCP are ensheathed by macroglial cells, i.e., astrocytes and Müller cells, contributing to the formation and maintenance of the inner blood-retinal barrier. While the astrocytes’ bodies and processes are found exclusively in the nerve fiber layer, Müller cells’ bodies are located in the inner nuclear layer, and they project processes through entire retinal thickness [28]. The high density of aquaporin-4 expressed on these cells is targeted by disease-specific IgG under inflammatory conditions. The T cells get access to the retina from SCP and DCP and open the BRB for the AQP4-IgG and complement. It was experimentally shown on an animal model that retinal damage may appear independently of optic neuritis [29, 30].

Our study’s limitation was a small group of NMOSD patients due to the low prevalence of the disease. Overall, 24 patients with AQP4-IgG seropositive NMOSD were diagnosed in our department. However, at the beginning of study enrollment, we had to exclude some patients who could not undergo ophthalmic examination because of visual (n = 4) and physical disability (n = 3). The four patients refused to participate in this study. Therefore, further studies with larger cohorts are necessary to confirm our observations.

In conclusion, our findings suggest that in the eyes of MS patients, the vascular changes are secondary to the atrophied ganglion cell layer, while in the eyes of NMOSD patients, vasculopathy seems to be a primary process.
